# A retrospective analysis of perioperative complications associated with retropubic tension-free vaginal tape in 960 women

**DOI:** 10.1007/s00404-021-06299-x

**Published:** 2021-10-28

**Authors:** Janosch Jahn, Christl Reisenauer, Birgitt Schoenfisch, Bastian Amend, Sara Yvonne Brucker, Juergen Andress

**Affiliations:** 1 Department of Gynecology and Obstetrics, University Hospital Tuebingen, Calwerstrasse 7, 72076 Tuebingen, Germany; 2 Department of Urology, University Hospital Tuebingen, Tuebingen, Germany

**Keywords:** Intraoperative complications, Postoperative complications, Stress urinary incontinence, Tension-free vaginal tape (TVT)

## Abstract

**Purpose:**

The purpose is to analyse perioperative complications associated with the retropubic tension-free vaginal tape (TVT) procedure and their management.

**Methods:**

This retrospective, monocentric cohort study included 960 women after retropubic TVT procedure performed by one surgeon from 2011 to 2016. Complications were identified up to 6 weeks after the procedure, divided into specific and general complications and classified based on the Clavien–Dindo (CD) Classification. A visit 6 weeks after the surgical procedure was attended by all patients.

**Results:**

77 complications, of which 74 occurred postoperatively and 3 intraoperatively, affecting 72 (7.5%) out of 960 women. Urinary retention and voiding problems were the most common complication. The mean age of women suffering complications was 3.4 years higher in comparison to the mean age of women without complications (*p* = 0.036). The Body Mass Index (BMI) of the group of women with perioperative complications had an average BMI which was 0.5 kg/m^2^ lower than the average BMI of the women without complications. 22 (12.8%) out of 172 women with recurrent stress incontinence had postoperative complications, of which 21 were related to the TVT.

**Conclusion:**

The retropubic TVT is a surgical procedure associated with a low number of perioperative complications, even in the group of elderly and overweight women, as well as in cases of recurrent stress incontinence.

## Introduction

Female urinary incontinence is a worldwide issue, with the incidence increasing with age [1]. Approximately 50% of women worldwide have to deal with this problem once in a lifetime [2] and will, therefore, lose quality of life [3]. Stress incontinence is a common subtype affecting 37% of women older than 40 years in Germany [4].

Non-surgical therapies should be considered first because they usually carry the least risk of harm [5]. Over the past years, the tension-free mid-urethral slings became the gold standard in the surgical therapy of stress urinary incontinence.

The retropubic tension-free vaginal tape (TVT) was first introduced by Ulmsten et al. in 1996 [6]. In comparison to earlier surgical treatments, the TVT supports the mid-urethra, is tension free and minimally invasive [7].

The retropubic TVT has already been subject of numerous studies. Many of them focused on specific parameters and complications in groups including only a smaller number of patients.

The aim of this study was to analyse the perioperative complications of retropubic TVT surgery and their management in 960 consecutive women operated on at one institution by one surgeon between 2011 and 2016.

## Methods

Women undergoing surgery with retropubic TVT between 2011 and 2016 carried out by one surgeon (CR) at the Department of Gynecology and Obstetrics Tuebingen were identified and their records were reviewed retrospectively. The operative reports, as well as the postoperative 6-week period, were explored. A visit 6 weeks after the surgical procedure was attended by all patients.

Data collected included baseline characteristics, the indication for surgery (stress incontinence or mixed incontinence with a predominant stress component), the presence of primary or recurrent incontinence, previous urogynecological surgeries, additional surgeries performed at the same time with the TVT procedure, type of anesthesia, operating time, type of the inserted retropubic TVT (TVT Exact®, Ethicon Inc., Johnson & Johnson, Somerville, NJ, USA or Retro-Arc®, AMS, Minnetonka, MN, USA), intraoperative and postoperative complications and their management as well as length of the hospital stay. Residual urine was defined as 100 or more ml immediately (up to 10 min) after voiding, starting with the first micturition after TVT surgery.

All patients were pseudonymized.

### Statistics

All statistical analyses were performed using R version 3.6.3. We show means with standard deviations (SD) and range or numbers with percentages as appropriate. Differences in age, BMI, operation time and hospitalization in patients with and without complications were tested using the *t* test, nominal variables were assessed by Fisher’s exact test. A significance level of 5% was chosen.

Institutional approval: Independent Review Board (IRB)—Eberhard Karls University Hospital: 923/2018BO2.

## Results

960 consecutive women underwent surgery with retropubic TVT between 2011 and 2016. The patient characteristics at baseline are summarized in Table 1 and the surgery related characteristics are detailed in Table 2.

**Table 1:** Patient characteristics at baseline and patient characteristics with perioperative complications

Characteristics	All patients (*n* = 960)	Patients with perioperative complications (*n* = 72)	Patients with no complications (*n* = 888)	*p* value
Age, years^a^	59.5 (12.3) [25.6–90.7]	62.7 (12.9) [37.0–88.1]	59.3 (12.2) [25.6–90.7]	0.036^b^
Body Mass Index, kg/m^2^^a^	27.1 (5.0) [17.2–49.7]	26.6 (4.3) [18.9–37.5]	27.1 (5.1) [17.2–49.7]	0.389^b^
Previous urogynecological surgery, *n*	354 (36.9%)	37 (51.4%)	317 (35.7%)	0.011^c^
Type of incontinence, *n*				0.518^c^
Stress incontinence	794 (82.7%)	62 (86.1%)	732 (82.4%)	
Mixed incontinence	166 (17.3%)	10 (13.9%)	156 (17.6%)	
Surgery indication, *n*				0.006^c^
Primary incontinence	788 (82.1%)	50 (69.4%)	738 (83.1%)	
Recurrent incontinence	172 (17.9%)	22 (30.6%)	150 (16.9%)	
Type of TVT, *n*				0.264^c^
TVT Exact®	882 (91.9%)	69 (95.8%)	813 (91.6%)	
Retro-Arc®	78 (8.1%)	3 (4.2%)	75 (8.4%)	
Combined procedures, *n*	102 (10.6%)	16 (22.2%)	86 (9.7%)	0.002^c^

^a^Data are characterized as median (SD) and range

^b^*t* test

^c^Fisher test

**Table 2:** Surgery-related characteristics

Characteristics	All patients (*n* = 960)	Patients with perioperative specific complications (*n* = 72)	Patients with no complications (*n* = 888)	*p* value
Operating time, min^a^	28.3 (17.3) [7–198]	33.2 (30.5) [15–98]	27.9 (15.7) [7–145]	0.147^b^
TVT	24.7 (6.7) [7–84]	23.8 (4.2) [15–38]	24.8 (6.8) [7–84]	
TVT combined with other procedures	58.6 (37.8) [16–198]	66.1 (53.3) [21–198]	57.2 (34.4) [16–145]	
Anesthesia, *n*				0.014^c^
Analgosedation	850 (88.5%)	56 (77.8%)	794 (89.4%)	
General anesthesia	107 (11.1%)	16 (22.2%)	91 (10.2%)	
Both	3 (0.3%)	0 (0.0%)	3 (0.3%)	
Hospitalization, days^a^	2.5 (1.9) [1–23]	5.4 (4.4) [1–23]	2.3 (1.3) [1–18]	< 0.001^b^
Single TVT	2.4 (1.6) [1–21]	4.7 (4.0) [1–21]	2.2 (1.1) [1–18]	
TVT combined with other procedures	4.0 (3.1) [1–23]	7.8 (5.2) [2–23]	3.3 (1.9) [1–12]	

*Min* minutes

^a^Data are characterized as median (SD) and range

^b^*t* test

^c^Fisher test

72 (7.5%) out of 960 women experienced perioperative complications, in three cases complications occurred intraoperatively, one of these women had an intra- and postoperative complication.

Complications were divided into specific TVT-related complications and general complications, which can occur following any surgery. All specific and general complications are listed in Table 3. Specific complications affected 63 out of 72 women (87.5%), whereas general complications were seen in nine women (12.5%).

**Table 3:** Intraoperative and postoperative complications

Complications	Specific complications (related to the TVT procedure)		General complications (can occur following any surgery)
Intraoperative complications, *n*	Bladder perforation (1) Bladder lesion (1)		Atrial fibrillation* (1)
Postoperative complications, *n*	Urinary retention and voiding problems (41) Urinary tract infection (10) Hematoma Retzius'’ space (4) Subcutaneous (1) Vaginal bleeding (4) Persistent stress incontinence (1) Increased urge component (1) Suprasymphysary pain (1) Suburethral vaginal erosion (1)		Heart problems Angina pectoris pain (1) Asystole (1) Atrial fibrillation* (1) Myocardial infarction (1) Hematoma in the right rectus abdominis muscle after coughing attack (1) Decrease of haemoglobin from 13 to 6.5 g/dl (1) Abdominal pain due to previously known abdominal adhesions (1) Diarrhea (2) Vaginal mycosis (1)

*The same patient had an atrial fibrillation intra- and postoperative

All complications were classified based on the Clavien–Dindo (CD) Classification and illustrated in Table 4 [8]. 37 complications were ranked as Grade III complications and required surgical intervention.

**Table 4:** Clavien–Dindo Classification of the perioperative complications after TVT surgery

a.* Intraoperative complications*			
Bladder perforation	1	The operation was continued	I
Bladder lesion of 5 mm length	1	The operation was stopped, the bladder lesion was sutured and after 5 days the TVT procedure was performed	IIIa
Atrial fibrillation*	1	The atrial fibrillation episode resolved spontaneously, and the operation could be continued	I

Specific (TVT-related) complications	Number of patients affected	Therapy	Clavien–Dindo Classification
b.* Postoperative complications*			
Residual urine and voiding problems	39	23 × TVT mobilization	IIIa
		1 × TVT mobilization twice	IIIa
		1 × TVT cutting after mobilization	IIIa
		1 × TVT cutting after mobilization twice	IIIa
		8 × indwelling catheter	II
		2 × indwelling catheter, pharmacological treatment	II
		2 × pharmacological treatment	II
		1 × observation	I
Residual urine + hematoma in the Retzius' space	1	TVT mobilization, mini-laparotomy hematoma removal and wound drainage	IIIb
Persistent stress incontinence + residual urine + urinary tract infection	1	Re-TVT procedure followed by TVT mobilization	IIIa
		Antibiotic therapy	II
Urinary tract infection	9	Antibiotic therapy	II
Hematoma in the Retzius' space	3	Mini-laparotomy, 2 × additionally wound drainage	IIIb
Suprasymphysary subcutaneous hematoma	1	Observation	I
Vaginal bleeding	4	1 × wound revision (re-suturing)	IIIa
		3 × vaginal package and pause of anticoagulants	I
Suprasymphysary pain	1	Symptomatic treatment, (analgesics)	II
Increased urge component	1	Anticholinergic drugs	II
Suburethral vaginal erosion	1	Local excision	IIIb
General complications			
Hematoma in the right rectus abdominis muscle after a coughing attack + decrease of haemoglobin from 13 to 6.5 g/dl	1	Transfusions and exclusion of a continuous bleeding by Angio CT and laparoscopy	IIIb
Angina pectoris pain	1	Transfer to the intensive care unit and symptomatic treatment	II
Asystole over 20 s in the recovery room	1	10 s. resuscitation, transfer to the intensive care unit	I
Atrial fibrillation in the recovery room*	1	Transfer to intermediate care unit, application of potassium and magnesium	II
Myocardial infarction	1	Transfer to internal medical department	IIIa
Diarrhea	2	Conservative therapy	I
Abdominal pain due to previous known abdominal adhesions	1	Laparoscopic adhesiolysis	IIIb
Vaginal mycosis	1	Antimycotic local treatment	II

*The same patient had an atrial fibrillation intra- and postoperative

Two intraoperative specific complications occurred, a bladder perforation and a bladder lesion of 5 mm length. The latter occurred during preparation of the paraurethral tunnel. In the case of bladder perforation, the surgery could be continued. The TVT needle was removed and inserted new. The cystoscopy following the procedure was without findings. A transurethral catheter was applied, and the woman stayed 2 days in the hospital for observation. In the case of the 5 mm bladder lesion, the bladder wall was sutured, and an indwelling catheter was left in place for 5 days. At the same time the catheter was removed, a second TVT procedure took place, successfully.

Postoperative 41 women showed voiding problems and residual urine. In 25 women the TVT was mobilized once, in 2 woman the TVT had to be mobilized twice of which one tape had to be cut afterwards. In one further case the TVT was cut after a single mobilization. Ten women had an indwelling catheter, of who two additionally received a pharmacological treatment. Two women only received a pharmacological treatment and in one woman the voiding problems resolved spontaneously. The affected patients showed a functional recovery after treatment.

In one woman the stress urinary incontinence persisted, so that the TVT was removed and a new one was inserted on the second postoperative day.

Hematomas in the Retzius' space occurred in four women and all had to be managed surgically by laparotomy and removal of hematoma. The hematomas were detected on regular postoperative ultrasound.

Urinary tract infections affected ten women who were all treated with antibiotics.

Four women complained about vaginal bleeding postoperatively, in one case the vaginal wound had to be re-sutured, the other cases were treated conservatively (vaginal package and intermittent pausing of anticoagulation).

A single suburethral vaginal erosion occurred. The tape erosion was excised.

A woman suffering from mixed incontinence experienced a slightly increased urge incontinence after surgery and received a pharmacological treatment. In a case of increased suprapubic pain, the woman was treated with analgesics and another woman with a suprapubic hematoma was treated conservatively.

## Discussion

In this retrospective study, the retropubic TVT has proven to be a surgical procedure with a low risk of perioperative complications. 77 complications, of which 74 occurred postoperatively and 3 intraoperatively, affected 72 out of 960 patients. Complication rates were low even in elderly and overweighted patients, as well as in women with recurrent stress incontinence.

In our study, 3 (0.31%) women suffered intraoperative complications, of which 2 were TVT related (one bladder perforation and one bladder lesion). Regarding the number of intraoperative complications, a wide range from 0.76 [6] to 16.58% [9] can be found in literature. Reported bladder injuries in connection with a TVT range from 1.79 to 5.8% [10–12].

The way how the intraoperative bladder injuries were handled matches with descriptions in the literature. If during the operation, a bladder perforation had been detected by cystourethroscopy the operation was continued in the majority of cases, and the patient was supplied with a catheter postoperatively [13, 14]. In case of an extended lesion the operation was stopped [10].

The types of specific postoperative complications which occurred, and their frequency are similar to those described in the literature [3, 6, 10, 15, 16]. Affecting 41 women, urinary retention and voiding problems were the most frequent complications in our study. This matches with reports from other studies were urinary retention and micturition problems totaled 52–77.7% [10, 15] of the occurred complications.

In comparison to previous studies reporting low numbers of urinary tract infections, making up less than 5% of all complications [14, 15] or not reporting a single one [10], our study showed ten urinary tract infections which make up 13.7% of the complications. Several further studies did not refer to urinary tract infections when discussing postoperative complications [16, 17]. The number of urinary tract infections seen in our study is still very low in comparison to the number of included women.

With 4 (0.42%) out of the 960 women suffering retropubic hematomas, our rate is slightly lower in comparison to previous studies, reporting 0.78–1.1% of the patients affected [10, 11, 14, 15].

Regarding the frequency of postoperative complications our rate of 7.3% affected patients is lower than in many other studies recording postoperative complications rates from 13 to 30% [9–11, 15, 16]. This is undoubtedly linked to the high expertise and experience of the surgeon. There are also studies numbering fewer postoperative complications. In one of the first studies concerning the retropubic TVT from Ulmsten et al. [6], 4.58% of 131 patients experienced postoperative complications. Possible reasons are the mean age of the patients—with 53 years, 6 years younger than patients included in our study—and the study design in which only women with primary stress incontinence were included.

The management of postoperative complications in the present study is comparable to procedures described in other studies. Cases of small residuals can be treated conservatively [18]. Additionally, an intermittent catheterization can be helpful [10, 11, 15, 16, 18]. In cases of persistent urinary retention and micturition problems the TVT often has to be mobilized [10–12]. In case of a lack of success the TVT can be incised or removed [10, 12, 18]. In our study 28 women (2.9%) needed surgical intervention for postvoid residual urine. In 26 of these the tape had to be mobilized. In two cases the tape had to be incised after previous tape mobilization. This coincides with previous literature saying approximately 2.4–3.5% of the women after the TVT procedure require tape mobilization [10]. Urinary tract infections are usually treated with antibiotics [18]. Hematomas in the Retzius’ space can be managed with a mini-laparotomy [10, 18] and with surgical drainages [12].

The baseline characteristics of the included patients are similar to those in other studies [10, 15, 19] which makes the comparison of our results possible.

Several variables differed from those of other studies. With 25 min the mean operative time (TVT only subgroup) is shorter than in other studies, with mean operative times ranging from 30 to 38 min [15, 17, 20, 21].

The mean age of the patients who were affected by complications is significantly higher (3.4 years) than the mean age of the patients with no complications. Regarding only the patients with TVT-related specific complications the mean age is 4.3 years higher. This is comparable with other studies in literature. Engen et al. [3] describes an increasing complication rate in the 6th and 7th decade in comparison to the 5th decade, with bladder perforation rising from 2.2% to 3.2%. Toosz-Hoobsen et al. [19] report a higher number of voiding difficulties with increasing age.

The BMI of the women suffering perioperative complications (26.6 kg/m^2^) was 0.5 kg/m^2^ lower than the BMI of the patients with no complications (27.1 kg/m^2^). This can be explained with obese women (BMI of ≥ 30 kg/m^2^) being represented below average and with no extreme obese women (BMI of ≥ 40 kg/m^2^) represented in the group of women with complications. In defiance of the high number of women in this study only 77 (8%) had a BMI of 35 kg/m^2^ or higher, while they nearly make up to 14% of the included women in other studies [22].

172 (17.9%) of the 960 included women received TVT due to recurrent stress incontinence. In 22 (12.8%) of the latter perioperative complications occurred in comparison to 50 (6.3%) out of the 788 women with primary stress incontinence.

We cannot underline the fact that recurrent stress incontinence is a risk factor for bladder perforation [14]. Nevertheless, recurrent stress incontinence is linked with a higher number of perioperative complications in general [23]. 102 women had simultaneous procedures at the same time with the retropubic TVT, of which 16 (15.7%) had perioperative complications. Eight of these women had urinary retention and voiding problems, of which one additionally complained about persistent stress incontinence. Two patients had a retropubic hematoma. A urinary tract infection, a persistent urge component as well as a bladder perforation occurred once. Three women suffered general complications.

Complications occur more often when TVT is combined with additional procedures (*p* = 0.002).

To minimize pain and the risk of uncontrolled movement, the TVT procedures are often performed under general anesthesia [24]. Viewing the results of our study we regard the analgosedation as good anesthesia for the retropubic TVT procedure when referring to the rate of perioperative complications. Women who had general anesthesia suffered more often from perioperative complications than those with analgosedation. It must be mentioned that in our study only 107 out of the 960 patients (11.1%) were operated on under general anesthesia due to a combined procedure or women’s preference.

Finally, the choice of the anesthesia depends on the patients’ risk profile, the surgeons’ preference, and the expertise of the anesthetist [24].

Our study is limited by the retrospective study design. Also, an evaluation and comparison between the two inserted retropubic TVTs (TVT Exact® and Retro-Arc®) did not take place.

The strength of our study is the high number of women included as well as the experienced surgeon (CR) who carried out all surgeries. Furthermore, a detailed documentation took place intraoperatively and early postoperatively as well as at the visits 6 weeks after the surgical procedure which all patients attended.

The results of this study can be used to optimize patients counseling about the perioperative risks associated with the retropubic TVT.

## Conclusion

The results of the study show that the retropubic TVT is accompanied by a low number of complications when performed by an experienced surgeon. We regard the retropubic TVT procedure under analgosedation as a very feasible surgical therapy for women affected by stress urinary incontinence or mixed incontinence with a dominant stress component. In spite of slightly higher complication rates in older and overweighted patients as well as in patients with recurrent stress incontinence, the overall number of complications was low.

## Data Availability

All data are available (corresponding author).
